# Formal Psychological Assessment in Evaluating Depression: A New Methodology to Build Exhaustive and Irredundant Adaptive Questionnaires

**DOI:** 10.1371/journal.pone.0122131

**Published:** 2015-04-15

**Authors:** Francesca Serra, Andrea Spoto, Marta Ghisi, Giulio Vidotto

**Affiliations:** Department of General Psychology, University of Padua, Padua, Italy; University of Rome, ITALY

## Abstract

Psychological Assessment can be defined as a complex procedure of information collection, analysis and processing. Formal Psychological Assessment (FPA) tries to improve this procedure by providing a formal framework to build assessment tools. In this paper, FPA is applied to depression. Seven questionnaires widely used for the self-evaluation of depression were selected. Diagnostic criteria for major depressive disorder were derived from the DSM-5, literature and Seligman’s and Beck’s theories. A Boolean matrix was built, including 266 items from the questionnaires in the rows and 20 selected attributes, obtained through diagnostic criteria decomposition, in the columns. In the matrix, a 1 in a cell meant that the corresponding item investigated the specific attribute. It was thus possible to analyze the relationships between items and attributes and among items. While none of the considered questionnaires could alone cover all the criteria for the evaluation of depressive symptoms, we observed that a set of 30 items contained the same information that was obtained redundantly with 266 items. Another result highlighted by the matrix regards the relations among items. FPA allows in-depth analysis of currently used questionnaires based on the presence/absence of clinical elements. FPA allows for going beyond the mere score by differentiating the patients according to symptomatology. Furthermore, it allows for computerized-adaptive assessment.

## Introduction

The increase of depression in the last few years is a debated topic [1–5]. Some authors argue that, nowadays, depressive disorders with bipolar disorder are the most common type of disease in the world, though often unrecognized and inadequately treated [2–4].

The correct identification of depression during the assessment phase is a critical issue. In general, the quality of the clinical evaluation is always fundamental for diagnosis and treatment. An incorrect psychological assessment may result in patients’ dissatisfaction and suffering [6].

The assessment can be defined as a complex procedure of information collection, analysis, and processing [7]. The main tools available to carry out an assessment are classifiable into four categories [6]:

Clinical interview and observation provide a large amount of information, follow adaptive logic, and use multiple channels, but they need a great deal of time to be completed. The clinician may also introduce inference problems.
Psychophysiological measurement provides objective data and deeply assesses aspects that cannot be evaluated in other ways; nevertheless, it is limited in terms of the areas of application, it is sometimes inaccessible, and can be affected by artifacts.
Self-report questionnaires allow for the systematic and quick collection of a large amount of information and avoid embarrassing patients; on the other hand, they redundantly (non-adaptively) investigate constructs and provide a quantitative numeric score that does not systematically account for qualitative information.
(Semi) structured interviews are of great importance. Structured interviews are similar to orally administered questionnaires [8], whereas semi-structured interviews [9,10] do not follow a predetermined sequence of questions, since the questions’ order depends on the previously collected answers. Semi-structured interviews introduce the crucial concept of *adaptivity*, which plays a central role in this paper. For clinical interviews, however, they need a great deal of time for their completion, and the clinician may introduce inference errors.

Overall, the salient characteristics of an ideal assessment tool for the initial evaluation are as follows: it should be as time-consuming as a self-report questionnaire, as suited to collecting information as an interview, adaptive, valid, reliable, and encouraging of correct logical inferences. *Formal Psychological Assessment* (FPA [7]) is an attempt to provide such an instrument.

### Formal Psychological assessment

The FPA is a new methodology potentially capable of maximizing the advantages of both semi-structured interviews and self-report questionnaires by overcoming the limitations of these tools and managing the problems of traditional assessment [7].

The ability to analyze clinical symptoms is important when evaluating the responses to a questionnaire. FPA goes beyond the score of the patient and investigates the diagnostic features implicated by the responses. The crucial issue that represents the starting point of FPA is consideration of the information that can be collected from a patient’s numeric score on a questionnaire. For instance, if a nine dichotomous items scale is administered to a patient and the clinical cut-off score of the scale is 7, there are 46 different clinically significant response patterns (one pattern with score 9, nine patterns with score 8, and 36 patterns with score 7). Each of these patterns may convey clinically different information about the patient. Notice that all this information is already included in the questionnaire, even if the mere score somehow hides it. Nevertheless, at the present time, the only ways the clinician has to account for the specific information endorsed by the pattern are: i) to read all the items the patient has answered affirmatively, and from them, try to deduce his/her clinical situation (it is noteworthy that this solution is applicable only when the questionnaire counts a low number of items, and that this operation cannot be carried out when tolls like the MMPI-2 are administered); and ii) to further investigate these issue through psychological interview. Both of these solutions do not provide any standardized procedure comparable to the systematic scoring of the questionnaire. The FPA aims to provide an in depth analysis of the specific response pattern observed, thereby informing the clinician about the actual diagnostic configuration of the patient at hand. This opportunity is assured by an a priori analysis of the clinical elements investigated by each of the items of the questionnaire. Such analysis is the deterministic skeleton on which it is possible to implement a probabilistic adaptive procedure capable of mimicking a semi-structured interview within the frame of a questionnaire. By highlighting the specific clinical elements investigated by each single item of a questionnaire, FPA highlights the differences among patients that would otherwise be hidden by the simple score. From a clinical perspective, it allows for an idiographic and nomothetic diagnosis.

The FPA is the formal conjunction and clinical application of two theories of mathematical psychology, the *Knowledge Space Theory* (KST) [11–13] and the *Formal Concept Analysis* (FCA) [14,15], and is intended to build a formal representation of the relationship between the items of a questionnaire and a given set of diagnostic criteria. In FPA, each item included in a clinical self-report questionnaire (or interview) is defined as an *object*. Each object can be described on the basis of a set of elements referring to a given theoretical framework. Such elements, (which can be either clinical symptoms or the decomposition of the diagnostic criteria used to specify one or more clinical disorder), are named *attributes*. Thus, each object can be related to the set of attributes it endorses. For instance, in characterizing the items (objects) of a given clinical self-report questionnaire, the attributes may be represented by the DSM-5’s diagnostic criteria of disorder the questionnaire is supposed to investigate.

Theoretical flexibility is one of the major strengths of the FPA. In fact, the same objects can be described in terms of attributes by referring to different appropriate frameworks [7]. Each item may investigate one or more attributes and each attribute can characterize one or more items. For example, the item “I am less interested in sex than I used to be,” related to depression, investigates the attributes “Diminished interest and pleasure” and “Decreased interest in sex,” which represent two diagnostic criteria for depression in the DSM-5. On the other hand, the attribute “Diminished interest and pleasure” is investigated by several items (i.e., “I have lost most of the interest in other people or things”, “I am less interested in sex than I used to be”, “I do not want to do anything” and “I seem to have lost interest in the future”).

Starting from a set of items and a set of attributes, a *Boolean matrix* can be constructed assigning to each object its own set of attributes. Every time an item investigates a specific attribute, the corresponding cell of the matrix will contain “1,” otherwise the cell will contain “0.” In FPA this matrix represents the *clinical context*. The entire set of objects is the *domain* of the clinical context. The *clinical state* of a patient consists of the subset of items he/she answered affirmatively. It is noteworthy how each clinical state (depicted by the response pattern endorsed by a patient) is defined by a unique subset of attributes. Thus, even if two patients respond affirmatively to the same number of items (i.e., obtain the same score to the questionnaire), the representation of their two states in terms of attributes are systematically different, if the items affirmatively answered are also different. Thus, different states may have the same clinical score [7], but will collect different attributes. This is fundamental from a clinical point of view, since it allows for the analyzing and, therefore, the treating, of each subject individually, according to his/her symptoms configuration. The clinical context is the Boolean representation of the *clinical structure*, which is the set representation of the implications among the items of the domain. The clinical structure contains all the clinical states that are formally expressed by the matrix. In fact, not all the subsets of items are admissible response patterns given a theoretical framework (thus, given the formal context). For instance, if a given item *i* endorses attribute *a*, while item *j* endorses attributes *a* and *b*, the clinical state corresponding to *{i}* is admissible. On the other hand, the state *{j}* is not admissible since a person who affirmatively answers item *j* is supposed to present both attributes *a* and *b*, and thus, he/she should affirmatively answer even item *i*. For this reason, the state *{i*,*j}* is admissible too. In this case, item *i* is said to be a *prerequisite* of item *j* since there is no state in the structure that includes the latter but not the former. The prerequisite relation among the items, obtained from the matrix through the formal mathematical passages, can be represented as a complete lattice depicting the clinical structure.

To sum up, the first step of FPA methodology is the deterministic model construction, which consists of the construction of the matrix assigning to each item of the scale the subset of attributes it investigates. The second step concerns the construction of the clinical structure from the attributes assignment. The result can be represented as a lattice where each node represents a clinical state and its set of attributes [16]. The lattice is a deterministic representation of the prerequisite relation among the items of the domain. It is evident how a completely deterministic approach is inadequate for assessment in clinical practice for two main reasons: first, not all clinical states have the same probability of occurring; second, in self-report tools, problems with patient insight or with item wording may prevent a perfect correspondence between the observed response pattern and the actual clinical condition of the patient. Therefore, a probabilistic approach is needed. The basic local independence model (BLIM) [12] is a probabilistic model that defines a probabilistic clinical structure where a probability value is assigned to each clinical state. Under BLIM, the responses to each item are locally independent given the clinical state of a subject. Starting from the probabilistic structure, the probability of a response pattern depends on the conditional probability of that pattern given an underlying clinical state (for each state; [11]). The conditional probability is defined by the false negative and the false positive rates for each item [12–13].

The clinical structure, by means of the probabilistic weights obtained through the application of the BLIM, could be used to implement an algorithm for an adaptive, quantitative and qualitative tool: adaptive because, based on the structure, it selects each question to maximize the collectable information; quantitative because it could provide a numerical score; and qualitative because it provides information about all the subjects’ symptoms.

The present paper aims to describe a practical application of FPA to illustrate procedural issues, discuss the advantages of the approach, and show its potential for psychological assessment in this case, relating to depression.

## Materials and Methods

In this study we analyzed the relations among a large set of items used to investigate depression through self-report measures and a set of attributes that mostly refer to three areas: the Diagnostic and Statistical Manual of Mental Disorders, fifth edition (DSM-5)[17], the clinical features most frequently reported in literature and Seligman’s and Beck’s etiopathogenetic theories.

It is known that depression is characterized by deep sadness and despair, hopelessness, helplessness, and worthlessness [18–20]. Furthermore, a depressed mood is associated with anhedonia [18, 21], apathy [22–24], loss of motivation [25], crying [18,26], and irritability [27,28]. Feelings of guilt are frequent [18,29] and, in more severe forms, they can result in delusion of guilt [18,30]. Sleep problems characterize depressed patients and frequently their insomnia is “terminal” (waking early in the morning) or characterized by frequent nocturnal awakenings or by feelings of not being rested after waking up [18,31,32]. Work and social relationships are often severely compromised [18,33]. Psychomotor retardation can present as simple motor slowing, but more often does so as ideation and speech slowing as well as concentration difficulties [18,34]; accompanying this is fatigue and energy loss [35]. Many patients experience agitation, which can manifest as restlessness, incapacity to sit still, torturing hands and/or hair or even biting nails and/or lips [26]. Sexual disorders such as decreased libido can be observed [18]. Lastly, ideas of death such as “Life is not worth living” are usually associated with depressed mood; the patient wishes to die or thinks, plans, carries out attempts and, relatively frequently, dies by suicide [18,36,37]. Suicide is the most tragic consequence of depression and the number of suicides has not decreased since the clinical use of antidepressants [38] but increased with the crisis [39–41].

Beck’s and Seligman’s theories need further explanation. Beck’s model [42–46], considers the dysfunctional schemes to be rigid, impermeable and absolute as distorted representations of experience. They influence the interpretation, identification, categorization and evaluation of experience. Beck [47] categorizes typical beliefs and mistakes of depression as a *cognitive triad* that includes a negative view of self (“I am a failure”), a negative view of the world (“the world is evil and unhappy”), a negative view of the future (“I’ll always be a loser”). Seligman’s theory [48], based on animal experimentation, suggests that depression is associated with the conviction that nothing can be done to face stressful life events [49]. This is *learned helplessness*, which tends to be generalized to new situations with the expectation of having no control over the future [50–52].

In the research we explored the attributes derived from DSM-5 diagnostic criteria for major depressive disorder, Seligman’s and Beck’s theories and, finally, attributes widely described in the literature (such as apathy [22–24] and irritability [27,28]). Subsequently, tools and clinical symptoms of depression have been selected for the construction of the model according to FPA procedure; they are presented in the next section.

### Self-report Questionnaires and Attributes

Four self-evaluation questionnaires developed in English, one self-report questionnaire in French, and two self-report questionnaires in Italian were selected (each here presented in English).

The *Beck Depression Inventory II* (BDI-II) [53] is one of the world’s most widely used self-report questionnaires for the evaluation of depression. It appears to be both agile and sensitive. Each item has four possible answers of increasing severity. BDI-II contains 21 items that explore various facets of depression.

The *Self-rating Depression Scale* (SDS) [54] assesses the level of depression. It consists of 20 items that explore affective (2 items), somatic (8 items), and psychological (10 items) aspects of depression. The tool is very simple and quick.

The *Rome Depression Inventory* (RDI) [55] consists of a series of 25 items that use the phrases most frequently used by depressed patients to describe their illness and discomfort.

The *Plutchik-Van Praag self-report depression scale* (PVP) [56] was developed with 34 items to cover all the DSM-III diagnostic criteria for depression. Since these diagnostic criteria have remained largely unchanged in DSM-5, this scale still holds great validity.

The *Carroll Rating Scale* (CRS) [57], is Carroll and Feinberg’s self-report version of the Hamilton Rating Scale for Depression (HAMD) [58], consisting of 52 dichotomous items.

The *Self-Assessment Scale for Depression* (SAD) [59], whose authors tried to use a language close to that of patients in the formulation of the items to contribute to a better comprehension of questions and a higher reliability of the instrument, consists of 31 items.

Finally, the *Center for Epidemiological Studies Depression* (CES-D [60,61]) has been one important instrument in depression epidemiology since its first use in Community Mental Health Assessment Surveys in the 1970s. The self-report version is widely used and consists of 20 items.

In conclusion, the total number of items adds up to 266.

Table 1 displays all the attributes considered to assess depressive symptomatology: diagnostic criteria from the DSM-5, Beck’s and Seligman’s theories and other information from the literature review.

**Table 1:** The twenty attributes of the clinical context.

Attribute	Explanation
**A1**	Depressed mood
**A2**	Diminished interest and pleasure
**A3**	Decreased interest in sex
**A4**	Increase or loss of weight
**A5**	Gain or loss of appetite
**A6**	Insomnia or hypersomnia
**A7**	Agitation
**A8**	Psychomotor retardation
**A9**	Fatigue or energy loss
**A10**	Feelings of worthlessness (or Beck’s negative view of self)
**A11**	Feelings of guilt
**A12**	Diminished ability to think and concentrate
**A13**	Indecision
**A14**	Recurrent thoughts of death
**A15**	Suicidal ideation or attempted suicide
**A16**	Beck’s negative view of the world
**A17**	Beck’s negative expectation of the future
**A18**	Seligman’s learned helplessness
**A19**	Irritability
**A20**	Apathy

### Procedure

Every item in the clinical self-report questionnaires described above was initially considered. These items became the objects of the matrix and represented the rows of the matrix for an initial total of 266 items (BDI-II: 21x4, SDS: 20, RDI: 25, PVP: 34, CRS: 52, SAD: 31, CES-D: 20). The attributes of the clinical context were obtained from the DSM-5 (15), Beck’s theory (2), Seligman’s theory (1), and the literature (2) for a total of 20 attributes, which are placed in the matrix columns. In this way it is possible to find out what attributes belong to each item, what attributes describe any particular object, and to identify relationships of great clinical and formal importance among objects and attributes. Two experts in the field of depression built the clinical context (i.e., the Boolean matrix).

Applying the FPA, four different configurations that may occur within the matrix deserve a separate description, since they produced important modifications in the number of items to be included in the final model:

1. Items that investigate none of the attributes for depression are not useful for the measurement of the construct (their row in the matrix will contain only zeroes).
2. Different items investigating the same set of attributes form equivalence classes. It is then useful to choose the items that relate better with the investigated attributes.
3. Attributes not investigated by any item necessitate the construction of new ad hoc items to investigate them.
4. Some items present problems with phrasing, construction, or validity.

The resulting formal context is the starting point for the construction of the clinical structure and the structure is the reference point for the adaptive algorithm calibration of FPA (the description of which goes beyond the aims of the present paper).

## Results

The clinical context is the first result presented. Therefore, from Table 2, it can be seen that the first key result is that we were able to get 30 equivalent classes collecting the same information redundantly investigated by the initial 266 items. Conditions 1, 2, and 4, described in the previous section, allowed us to group many questions repeated with different words, in the various self-report questionnaires consulted; some items were eliminated since they did not investigate any of the selected attributes; the two professionals excluded others because of problems with their phrasing (double sentences, fuzzy adverbs). Through these procedures, we were able to get a much more malleable matrix with 30 equivalent classes covering all the identified diagnostic criteria**.**

**Table 2:** The clinical context containing the thirty items and the twenty attributes.

ID	Item	Text	Attributes
I1	BDI-II.24	I feel like I am being punished	A 11
I2	BDI-II.42	I feel more restless or wound up than usual	A7
I3	BDI-II.47	I have lost most of the interest in other people or things	A2, 20
I4	BDI-II.51	I have much greater difficulty in making decisions than I used to	A13
I5	BDI-II.58	I have less energy than I used to have	A9
I6	BDI-II.63	I sleep a lot more than usual	A6, 8, 9
I7	BDI-II.74	I can’t concentrate as well as usual	A12
I8	BDI-II.82	I am less interested in sex than I used to be	A2, 3
I9	ZUNG.1	I feel down-hearted and blue	A1
I10	ZUNG.3	I have crying spells or feel like it	A1, 7
I11	ZUNG.4	I have trouble sleeping at night	A6
I12	ZUNG.15	I am more irritable than usual	A 19
I13	ZUNG.19	I feel that others would be better off if I were dead	A14, 10 11
I14	RDI.4	I do not really want to eat	A5
I15	RDI.7	I do not want to do anything	A2 9
I16	RDI.8	I seem to have lost interest in the future	A2, 17, 20
I17	RDI.10	I feel quite useless	A10
I18	RDI.23	I feel a burden to others	A10, 11
I19	PVP.23	The speed of my thinking seems to be reduced	A8, 12
I20	PVP.28	I think of the families and friends who have died	A14
I21	PVP.31	I have made a suicide attempt	A15
I22	CRS.2	I am losing weight	A4
I23	CRS.12	Dying is the best solution for me	A14, 15
I24	CRS.19	I wake up often in the middle of the night	A6, 7
I25	CRS.21	I am so slowed down that I need help with bathing and dressing	A8, 9, 20
I26	CRS.47	I get hardly anything done lately	A 8, 9, 18
I27	CRS.48	There is only misery in the future for me	A 17
I28	SAD.30	I have the impression of being aloof and not feeling affection for my family members	A 20
I29	CES-D.3	I felt that I could not shake off the blues, even with help from my family or friends	A1, 18
I30	CES-D.15	People were unfriendly	A16

^a^ This representation, which is convenient for explanatory purposes, is equivalent to the Boolean matrix with thirty rows and twenty columns introduced in the paper.

None of the explored questionnaires could cover all the attributes for depression alone, however. BDI-II does not provide information concerning Beck’s negative view of the world and learned helplessness. In SDS, there are no items investigating psychomotor retardation, possible feelings of guilt, possible suicidal attempts, or thoughts and learned helplessness. Some symptoms are part of the diagnostic criteria of the DSM-5 and, despite their obvious importance, are not considered. Even in RDI, some of the attributes derived from the DSM-5 are not investigated: weight modifications, the decrease in sexual interest and pleasure, psychomotor retardation, ideas and possible suicidal attempts and learned helplessness. While PVP was built to create an ad hoc self-report questionnaire for depression investigating all DSM diagnostic criteria, it does not take into account negative view of the world, negative expectation for the future, or learned helplessness. The only attribute missing in CRS is indecision. SAD does not take into account psychomotor retardation and suicidal ideation or attempts. Finally, the CES-D does not investigate decreased interest in sex, change in weight, indecision, recurrent thoughts of death, suicidal ideation or attempts or irritability.

Another interesting result of the application of FPA to the set of items was the identification of several items with methodological problems such as *double phrases* (“I’m depressed” *or* “I often want to cry”), *fuzzy adverbs* (“my life is *pretty full*”), or problems with *content validity* (item CRS.40: “I got sick because of the bad weather we have been having”).

Another important key finding is that the matrix allowed analysis of the equivalent classes of items and their attributes: some classes investigate subsets of attributes assessed by others. In this way a prerequisite relationship among different classes is derived. For instance, RDI-10, “I feel quite useless,” which investigates feelings of worthlessness, is a subset of RDI.2, “I feel a burden to others,” which also contains guilt; and these two items are prerequisites for SDS.19, “I feel that others would be better off if I were dead,” which contains feelings of worthlessness and guilt, and thoughts of death.

The relationships created among items in the matrix generate the clinical structure.

Many other inclusion relations among equivalent classes were observed and can be derived from Table 2. All these relations are critical because they describe FPA’s adaptive reasoning, suggesting the possibility of applying prerequisite relations in the clinical context. In fact, if the response to the prerequisite of a particular item is negative, a positive response to the other item will be logically excluded. The whole set of prerequisite relations (depicted by the clinical structure) could be implemented into an algorithm for applying an adaptive assessment using the items of the investigated questionnaires.

An important observation concerns RDI.2, “I feel better in the evening than in the morning.” It is difficult to assign attributes to this item, but despite this it is very representative of depressive symptoms. Perhaps it would be appropriate to create an ad hoc attribute for this item.

Finally, in all 266 items investigated, some items investigate the same attribute (feelings of worthlessness), but have different facets. For example, the BDI-II-12, “I feel I am a total failure as a person,” BDI-II-27, “I am disappointed in myself,” and RDI-10, “I feel quite useless.” It might be interesting to try to create different attributes for each facet.

## Discussion

This paper has shown how the FPA highlights each self-report questionnaire’s strengths and weaknesses in terms of correspondence to a set of diagnostic and clinical criteria. The FPA details the relations between objects (items) and attributes (decomposition of clinical and diagnostic criteria). This eliminates useless redundancy and increases efficiency. FPA also allows for the pinpointing of the relations among sets of items and attributes by analyzing the presence or absence of diagnostic criteria in the items. Flexibility is another crucial advantage of FPA: the set of attributes could be easily modified or updated according to new versions of DSM or to different theoretical approaches, while the methodology remains equally effective and reliable. Moreover, it has been shown that FPA could actually be used to provide clinicians with the specific diagnostic information endorsed by the set of responses of a patient to a questionnaire. In FPA the score becomes somehow useless given that even smallest sets of items may endorse more critical situations in terms of clinical symptoms. All this information could be provided by means of the output of the adaptive algorithm that carries out the administration of the items.

One important result of the paper is the identification of 30 equivalent classes representing the basis for an assessment tool for depression. Such an instrument would explore all the selected diagnostic criteria in term of attributes, without redundancy, and would provide the clinician with a clear reference between items and construct criteria to mimic the interview procedure. This instrument should be validated in order to test its clinical structure. This goal, when reached, would, in turn, be a stepping-stone to the implementation of an adaptive algorithm for the assessment of depression, fulfilling the potentiality of FPA.

The strong innovation of FPA comes from the construction of the matrix that allows for the identifying of the actually existing relations among items in terms of the clinical symptoms they endorse. As stated before, such information is already present in the items, but it is hidden by a classical testing methodology that considers the score the most relevant output a questionnaire is supposed to provide with the clinician. The matrix can be expressed in terms of the clinical structure that is the core of the methodology. The structure is the set representation of the implications among the items of the domain (it contains all the clinical states). The prerequisite relation allows for adaptivity, just as, in a semi-structured interview, the individual is driven to respond to items according to what he answered previously. For example, in the case of depression, if a patient answers “no” to an item relating to “thoughts of death,” the algorithm will not investigate whether he intends to die by suicide because “having thoughts of death” is a prerequisite of the intention to die by suicide. In this way the tool becomes adaptive because it allows for a thorough analysis of the areas in which the patient suffers. This structure implies multiple advantages: 1) it avoids redundancy and unnecessary collection of information; 2) it saves time and energy; and 3) the clinician obtains qualitative information about a patient’s symptoms in a systematic and methodologically solid framework. Therefore, different response patterns (i.e., different attribute configurations) may characterize people who obtain the same scores on a self-report questionnaire. The algorithm, as well as FPA itself, will allow for discrimination between patients with the same scores but different symptoms. The information can be used by FPA to detect differences among these people, and produce specific indicators that could be used when planning treatments [62]. Specific psychological mechanisms underlying each patient’s phenomenology are thought to have implications for treatment effectiveness. Different combinations of symptoms could produce the same score on a self-report questionnaire, although such information might not be regarded in clinical practice. Indeed, considering two individuals who obtained similar scores on the Somatic-Affective Scale of BDI-II, such scores may arise predominantly from an elevation in either somatic or affective features. BDI-II does not allow for discrimination between the two cases. On the contrary, FPA is useful in clarifying the specific clinical configuration depicted by the observed response pattern, rather than the mere score. The opportunity to fruitfully use the qualitative information already present in the questionnaire, but hidden by the score, is crucial when it comes to suggesting the elective treatment strategies. Therefore, FPA could represent an important tool for improving case conceptualization and treatment implementation.

Despite the innovative perspective of FPA, it is important to note that in the present version, FPA shows some limitations, mostly related to the matrix construction process. This procedure is time consuming and the experts that undergo the task are prone to errors when following it. Only a few indexes about the inter-rater agreement are available in literature. Obtaining the matrix from a set of collected data, however, can reduce both time consumption and human error by the experts.

Summarizing, the FPA, through its methodology, allows for the construction of new clinical tools for clinical evaluation following efficient and effective principles beyond the assessment of depression. In this particular case, starting from several self-report questionnaires and numerous diagnostic criteria considered essential for the assessment of depression, the FPA applied to depression allowed for the creation of a potential tool with many added benefits compared to the self-report questionnaires used in the research. First, the 30 selected items allowed us to investigate all the diagnostic criteria selected for evaluation of depression; this was possible due to the construction of the matrix that made explicit the relationships between items (objects) and the decomposition of diagnostic criteria (attributes). The clinical structure gave us the opportunity to highlight the admissible response pattern (clinical states); the prerequisite relations highlighted by the clinical context, allow for the construction of an adaptive tool. This means we devised a tool that not only returns a quantitative score, but allows us to adaptively deepen the symptoms of the individual cases.

Overall, assessment by means of FPA can be quantitative, qualitative, and adaptive at the same time.
